# A Contribution to the Histo-Pathology and the Theory of Drug Eruptions

**Published:** 1907-05

**Authors:** 


### A CONTRIBUTION TO THE HISTO-PATHOLOGY AND THE THEORY OF DRUG ERUPTIONS.

As a result of a study along the lines indicated in the above title, Engman and Mook state their views as follows in regard to iodine and bromine eruptions:

The local eruptive phenomena are prone to occur at points of previous inflammation; about comedones, acne lesions, seborrheic lesions, scars, traumata, scratches, etc.

Traumata, pressure, quick changes of temperature may precipitate an eruption in tissues charged with the drug.

Idiosyncrasy or susceptibility may be admitted, as it is in other toxic conditions.

The glands or follicles of the skin take no active or specific part in the production of the lesions, and when they are involved it is secondary and passive to inflammatory changes about the vessels and in the connective tissue.

The gross histological changes in the skin consist in different degrees of inflammation, from slight changes about the vessels to destructive abscess formation and progressive death of tissue.

The minute histological changes or steps to this end may be classed in the following stages: First, increase of connective tissue cells about the vessel. Second, appearance of lymphoid-cells about the vessels. Third, addition to these cells of trifold leucocytes with granular appearance of collagen, and vacuolation of fixed connective tissue cells. Fourth, local increase of all these phenomena and the formation of an abscess.

The first and second conditions are found in the normal skin with iodide eruption.

The stages three and four, or the addition of leucocytes and degenerative changes, is induced by local disturbance of the normal equilibrium between the iodine combined in the serum and the tissues.

This disturbance of equilibrium may be induced by various factors, and when it does occur the resultant product acts as a toxin, which in turn causes tissue irritation and the production of various local inflammatory symptoms, the type of symptoms and the eruptions being dependent upon the character of the individual's reaction to the inflamation thus produced, as in any other toxic condition.

This theory may be termed the "rational theory," as it explains all the symptoms of iododerma and bromoderma in a purely rational, chemical, and mechanical manner, and does not depend in its elucidation upon the mysterious or purely theoretical action of the vasomotor system.—The Interstate Medical Journal, November, 1906.

